# Systems biology by the rules: hybrid intelligent systems for pathway modeling and discovery

**DOI:** 10.1186/1752-0509-1-13

**Published:** 2007-02-15

**Authors:** William J Bosl

**Affiliations:** 1 Harvard Medical School and Children's Hospital Informatics Program at Harvard-MIT Division of Health Sciences and Technology (ChIP@HST), Boston, MA 02115, USA

## Abstract

**Background:**

Expert knowledge in journal articles is an important source of data for reconstructing biological pathways and creating new hypotheses. An important need for medical research is to integrate this data with high throughput sources to build useful models that span several scales. Researchers traditionally use mental models of pathways to integrate information and development new hypotheses. Unfortunately, the amount of information is often overwhelming and these are inadequate for predicting the dynamic response of complex pathways. Hierarchical computational models that allow exploration of semi-quantitative dynamics are useful systems biology tools for theoreticians, experimentalists and clinicians and may provide a means for cross-communication.

**Results:**

A novel approach for biological pathway modeling based on hybrid intelligent systems or soft computing technologies is presented here. Intelligent hybrid systems, which refers to several related computing methods such as fuzzy logic, neural nets, genetic algorithms, and statistical analysis, has become ubiquitous in engineering applications for complex control system modeling and design. Biological pathways may be considered to be complex control systems, which medicine tries to manipulate to achieve desired results. Thus, hybrid intelligent systems may provide a useful tool for modeling biological system dynamics and computational exploration of new drug targets. A new modeling approach based on these methods is presented in the context of hedgehog regulation of the cell cycle in granule cells. Code and input files can be found at the Bionet website: www.chip.ord/~wbosl/Software/Bionet.

**Conclusion:**

This paper presents the algorithmic methods needed for modeling complicated biochemical dynamics using rule-based models to represent expert knowledge in the context of cell cycle regulation and tumor growth. A notable feature of this modeling approach is that it allows biologists to build complex models from their knowledge base without the need to translate that knowledge into mathematical form. Dynamics on several levels, from molecular pathways to tissue growth, are seamlessly integrated. A number of common network motifs are examined and used to build a model of hedgehog regulation of the cell cycle in cerebellar neurons, which is believed to play a key role in the etiology of medulloblastoma, a devastating childhood brain cancer.

## Background

A key issue in functional genomics is to understand how genetic or epigenetic perturbations to cellular machinery result in disease. Related to this is the question of how to perturb the system so that it functions in a desirable way. The first issue is the concern of hypothesis generation and the second is drug target discovery. Much of the work in these areas is now done by human experts who mentally integrate experimental data reported in the literature and perhaps statistical summaries of data from a variety of sources. Unfortunately, the number of papers published each year on any specific disease pathway continues to grow rapidly, making it difficult for specialists to incorporate all new results into their mental model of a pathway. Furthermore, the amount of data becoming available through new measurement technologies is far greater than the human brain can adequately synthesize. A principal goal of systems biology is to devise computer algorithms to help with the task of integrating expert knowledge, largely gleaned through published results, with high throughput data sources to create new hypotheses and putative drug targets. A particularly vexing problem is that the amount of data available about any pathway is usually incomplete. The human mind is fortunately very good at reasoning with incomplete knowledge, which enables researchers to create new hypotheses even when only incomplete information is available. Systems biology may benefit from emulating human reasoning: less detailed models that can incorporate many sources of incomplete information may yield more useful output [1].

Many engineering system designs today use descriptive models rather than mathematical models derived from mathematical formulas, such as differential equations. This is often achieved using a modeling approach based on fuzzy logic. Fuzzy logic is a computational method for formulating and transferring human expert knowledge to computational models. It provides a flexible tool for modeling the relationship between input and output information and is distinguished by its robustness with respect to noise and variations in system parameters [2-4]. This characteristic seems to mirror the robustness of biological systems and their remarkable ability to achieve precise functional control from imprecise components [5]. Fuzzy logic and several related, complementary software methodologies are sometimes lumped under the term 'soft computing' or hybrid intelligent systems. These represent a combination of emerging, complementary problem-solving technologies that include fuzzy logic, probabilistic reasoning, Bayesian networks, neural networks, and evolutionary or genetic algorithms. Models based on fuzzy logic are sometimes called rule-based models; these terms will be used interchangeably here.

### Fuzzy logic in medicine

Fuzzy logic has become ubiquitous in modern control systems engineering, including medical applications [6-9]. It has been particularly useful in applications where appropriate mathematical models cannot be derived due to the complexity of the problem. Examples include controlling the pump rate in artificial hearts and monitoring the hemodynamic state of patients during surgery [10], prediction of gait events using the electromyographic (EMG) activity of lower extremity muscles in children with cerebral palsy [11], pacemaker control [12], anesthesia and ventilator control [13], control of robotic prostheses [6], and numerous other medical [14] and engineering applications [6]. Perhaps one of the most immediately attractive features of fuzzy logic modeling for biology is that it provides a straightforward method for formulating and transferring human expert knowledge to quantitative computer models. Since much of the data about biological systems is derived from logically-designed, hypothesis-driven experiments and contained in linguistic form in journals, fuzzy logic provides a way for biologists to incorporate data that might otherwise be difficult to incorporate into computer models. Often this data is noisy and imprecise. Fuzzy logic models are naturally robust with respect to noise and variation in system parameters, but allow computation of logical consequences of complex system dynamics with imprecise variables.

At the same time, fuzzy logic models are capable of representing extremely complex systems to high degrees of accuracy when precise data is available. The standard additive model (SAM), a common formulation of a fuzzy logic system, is a universal approximator. That is, a SAM can approximate *any *nonlinear function as precisely as desired, give appropriate data [4]. Statistical and non-gradient based optimization methods such as genetic algorithms can be used to refine fuzzy logic models that are initially constructed by biologists manually or from pathway databases. Estimation of distribution algorithms, a type of evolutionary algorithm that replaces mutation and crossover operations with probability distributions [15], provide a potential way to use statistical high throughput data, such protein interaction data, to optimize manually constructed models or to provide putative hypotheses to fill in unknown network components. Thus, the methodology presented here, which is primarily focused on using expert knowledge to build computational models, can use the aforementioned soft computing and statistical methods to incorporate other data sources to optimize parameters, making it an ideal data integration tool. Since many textbooks and papers are available to describe fuzzy logic modeling details [6,9,16,17], the discussion here will focus on a novel implementation of a fuzzy logic system embedded in a network structure and its usefulness for spanning research in basic biology and clinical research.

### Fuzzy logic and qualitative methods for systems biology

Several related methods have been developed for fuzzy modeling of gene interaction networks from microarray data. The models are similar, but different methods are used to incorporate data and parameterize the models. Woolf [18] made fuzzy logic inference tables from triplets of genes to determine co-expression relationships. Sokhansanj, et al. [19] used an exhaustive search of gene interaction among twelve yeast cell cycle genes to fit the fuzzy model parameters. The model was able to predict transcriptional correlation among the twelve yeast cell cycle genes after tuning the model on a different data set for the same system. Linden, et al. [20] used a fuzzy logic model in a similar way to reverse engineer a gene interaction network from microarray data using genetic programming to discover and optimize the gene interaction rules. The goal of each of these models was to qualitatively represent coexpression patterns among genes.

Our goal is a broader modeling paradigm than the previous fuzzy logic applications. More along the lines of our goal are efforts to use qualitative differential equations (QDEs) to model biochemical dynamics in cells. Thus far this work has been primarily used for metabolic pathways. A modeling system that requires time series of metabolite concentrations as input data is described by King, et al. [21]. They used a QDE system to model ten reactions involved in glycolosis. Two distinct modeling tasks are identified: simulation of a biochemical system when model parameters are known and system discovery from time series data. For system discovery, machine learning methods from the soft computing paradigm were used to parameterize the QDEs. Time series of concentrations for proteins of known structure were used as input data to build the model, which required considerable computational time on a Beowulf cluster. One of the hindrances to adoption of this modeling approach is its computational representation that has evolved from the Lisp programming language.

### Heuristic reasoning for hierarchical complex systems

The methodology presented in this paper is distinguished from previous qualitative modeling approaches in several ways. The initial goal was to develop a model that would allow experimental biologists to use the kind of qualitative data found in typical journal articles to describe the interaction of genes, proteins, and other cellular components to create computer models of large numbers of interacting parts. This arose from a practical need in our research to keep track of myriad components in pathway models that were built from data extracted from dozens of journal articles. Biologists already do this kind of mental modeling every time they make a new hypothesis; a tool was needed to aid in this reasoning. Secondly, with new sources of data becoming available, it was important to design a methodology that could be expanded in the future to integrate new data sources to refine models.

Finally, biological processes span many scales. A kind of heuristic modeling is common in the literature, where molecular interactions are analyzed and used to create new hypotheses about cellular events, tissue processes or disease progression. For example, specific gene mutations accelerate tumor growth in specific tissues. This is a semi-quantitative relationship between two very different scales. Fuzzy network modeling can be used as a tool for aiding human reasoning when many interacting variables participate in complex interaction networks on several scales. Though the interactions can sometimes only be described approximately, the logic of the interactions is rigorous.

The modeling approach presented here was developed in an effort to create a computational tool that would emulate and extend the ability of human experts to create reasonable models from incomplete or ambiguous data. The goal was to allow these models to be constructed directly from the information contained in journal articles that typically report experimental results about molecular pathways. This approach adopts fuzzy logic for biochemical reaction modeling and all cellular processes and embeds this in a network structure. The network structure is similar to functional Petri nets [22-24], which are well-suited to representing molecular pathways and gene interactions, as well as physiological system interactions.

Pathway models can be constructed manually by biologists and manipulated to study the dynamics of alternative pathways. However, the power of this method is that it provides a framework for using various soft computing technologies to integrate diverse data sources to improve and refine models. Rule-based or fuzzy logic models [7,10,16,18,19] are appropriate for manipulation by genetic or other evolutionary algorithms, which may be useful for drug target discovery. This process will be discussed in future papers that expand the basic model presented here. Details about methods for integration of high-throughput data with expert knowledge will also be reserved for future publications. Because the soft computing paradigm has been widely adopted for many engineering tasks, it is hoped that the framework presented here can be adopted and rapidly expanded by many researchers with expertise in these methods. Input files and code for all examples presented are available at the Bionet website [25].

## Results and discussion

The most common childhood brain cancer is medulloblastoma (MB), a devastating disease that can result in permanent serious brain damage even when successfully treated [26-28]. The etiology of MB is believed to involve aberrant activation of Sonic hedgehog (Shh) signalling [27-31]. Potential therapeutic targets are therefore regulators of the hedgehog pathway [32-34]. Although much remains to be learned about the transcriptional hierarchy involved in hedgehog activation, enough experimental data is available to construct pathway models that are quite complex. Hedgehog regulation of medulloblastomas will be used as an example to illustrate our modeling approach.

**Figure 1:**
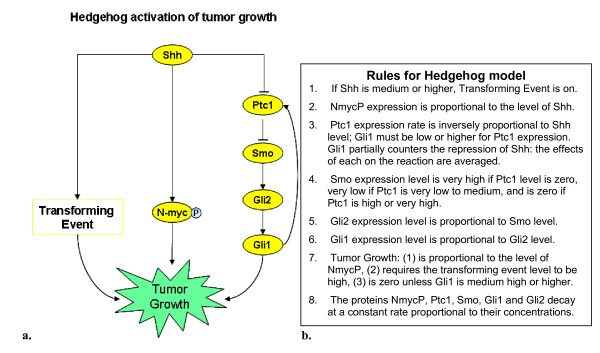
a. A schematic diagram of hedgehog regulation of tumor growth in the cerebellum is shown. These proteins are key components of the hedgehog signaling pathway, though many intermediate enzymes may mediate some steps. The "transforming event" is hypothesized to be required for tumor growth, but details about its function are completely lacking [61]. Tumor growth is a macroscopically defined process that is connected to molecular dynamics using rules. b. The rules for this model are given in linguistic form, much as they might be described in journal articles that discuss this pathway.

A model of hedgehog regulation of tumor growth will be constructed hierarchically, starting with a few molecular components and their role in tumor growth. Figure 1 presents the major hedgehog components that control "tumor growth". Tumor growth is a tissue-level quantity is regulated by a hierarchy of steps starting with Shh expression and hedgehog activation. Initially, we are interested in identifying the common network features or motifs that are involved in this system and building an extensible model that captures our understanding of the processes involved. The rules used to build this model are shown beside the figure. There are 8 nodes in this model and 15 reactions. Reactions for most variables include production and decay. Shh does not have production or decay reactions; it is set either to either its lowest or highest value in simulations. Specific motifs within this pathway will now be isolated and discussed for their relevance to the model. The rules for each of these cases are the same is for the whole pathway, except that lightly shaded parts are set to constant values.

**Figure 2:**
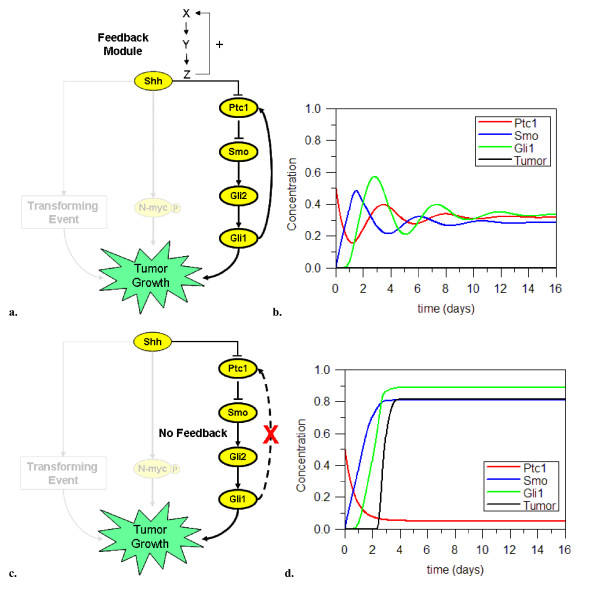
The important role of feedback loops is illustrated by isolating the Shh-to-Gli1 pathway in the hedgehog model. NmycP and the transforming event required for tumor growth are constitutively on in this simulation. a. The feedback from the transcription factor Gli1 to Ptc1 transcriptional activation is operative. As shown in b, Ptc1 transcription is maintained at an equilibrium level that is high enough to keep Gli1 below the threshold required for tumor growth. In c, the feedback is turned off; time courses in d show that increased Gli1 levels no longer influence Ptc1 expression, which is then effectively repressed by Shh. High levels of Gli1 are thus maintained and tumor growth is high. In this simulation Shh is constitutively at its highest level (0.1) and thus not shown.

Shh binds to the tumor repression gene Patched (Ptc1), releasing its suppression of Smoothened (Smo), which activates Gli transcription factors. The Gli transcription factors are the primary effectors of Shh signalling in the developing cerebellum [30,35]. Of particular interest is regulation of the Gli family of transcription factors that are believed to be involved in medulloblastoma tumorigenesis [26,28,29,33]. Several promising cancer drugs that reduce medulloblastoma growth in mouse models purportedly interfere with Gli1 and Gli2 expression by blocking the signalling pathway downstream of Smo. These include HhAntag [34] and cyclopamines [32,36,37]. A target of Gli1 is PTC1; Ptc1 is a transmembrane protein that blocks Smo expression when Shh binds to it. Gli1 activates Ptc1 expression and thus counters, to some extent Shh repression of Ptc1 activity [33,35,38]. The feedback from Gli1 to Ptc1 that is active in normal cells, as illustrated by figure 2, may play an important role in determining the effectiveness of drugs that regulate Gli1 expression downstream of Smo. Some reports suggest that this feedback loop may be inactivated in medulloblastomas *in vivo*, but not in cultured cells [33,34].

### Basic fuzzy network modeling concepts

We define a dynamic, interacting system by specifying the all of the variables that participate in the system and the processes (reactions) in which each node participates. The participating variables, also called nodes as in graph theory or Petri nets, can represent protein concentrations, gene expression levels, tumor size, temperature or any other variable of interest, including discrete variables.

Reactions or processes define dynamic changes to node values and must involve one or more nodes as reactants. Though the language used leans toward biochemical reactions, a useful feature of this rule-based model is the ability to connect dynamics on multiple scales. Nodes can represent any quantity for which rules of change can be defined. A reaction is defined by a reaction rate constant and a list of participating nodes together with their role in the reaction. The rate constant is a scaling factor that multiplies the reaction rate that is determined by changing reactant values. Reactions can also be defined as discrete events. Reaction rate has no meaning in this case; the event happens when the reactants satisfy the rule for firing and nothing happens when those conditions are not satisfied. This is useful for modeling processes such as chromosome separation or cytokinesis. Discrete events may also be a useful model for representing complex processes like metastasis as a single event that follows a long series of cellular reactions and sets into motion other processes.

For each reaction shown, the reactants have default rules based on their role as substrate, product, activator or inhibitor. These default rules can be overridden by giving new rules explicitly, as will be described in the methods section or may be optimized using automatic methods to integrate other data sources. The latter will not be discussed in detail here. A diagram of a system as in figure 1 is used to construct a computer model by first listing all of the participating nodes. Each reaction is then listed, with a reaction rate. Substrates are variables that are used up in a reaction; products are produced. Activators and inhibitors participate in a reaction, but their value doesn't change. The default rules for each of these roles are as follows:

• When a **substrate **is absent, the reaction rate is zero. As the concentration of substrate increases, the reaction rate increases proportionately. Thus, if the substrate level is low, its contribution to the reaction rate is low. The reaction rate, multiplied by the rate scaling factor or rate constant, determines how fast the substrate is used up in that reaction. By default, substrates have a stoichiometry of -1. This can be changed manually as needed.

• A **product **is produced in a reaction. It does not affect the reaction rate, except that when the product concentration reaches its highest allowable amount the reaction rate goes to zero. No more can be produced. The default stoichiometry is +1.

• **Activators **have the same default rules as a substrate: as the concentration increases, the reaction rate contribution increases. Activator concentration is not changed by a reaction in which it participates as an activator, it only influences the rate. Activators and inhibitors have a stoichiometry of 0, indicating that the reaction does not change their concentration.

• An **inhibitor **causes a reaction rate to slow as its concentration increases. When absent, an inhibitor has no effect on a reaction rate, which is then wholly determined by the concentrations of substrates and activators. As the amount of inhibitor increases, the reaction rate slows. The concentration at which an activator or inhibitor strongly affects a reaction rate can be set when known. By default, the effect is proportionate to the enzyme concentration.

In each reaction, the contributions of all participating reactants are averaged using one of several averaging schemes. We found that either a harmonic average or minimum rule works best. Both of these have the property that if the rule for any reactant makes the rate zero, the rate will be zero. Thus, if a reaction includes two substrates and an activator where the concentration of one substrate is zero, but the activator and the other substrate concentration are high, the output rule is zero: the reaction rate should be zero because one of the substrates is absent.

In rule-based simulations the meaning of "low" and "high" are determined by the dynamic range or universe of discourse for each variable. In the hedgehog model, most variables have a concentration range of [0.0, 1.0] for simplicity in illustrating the dynamics of particular motifs. However, the dynamic range of Shh is defined to be [0.001, 0.1] in this model. This was set to illustrate that variables can be assigned values in *any *desired range. Rules are defined based on the meaning of "low" and "high" within the context of the appropriate range for a given variable. Thus, a concentration of 0.1 is high for Shh in this model, while 0.1 is low for Gli1. In addition, the meaning of "low" and "high" for a given variable can be different in different reactions. Thus, in reactions where Gli1 is a product, such as a transcription reaction, low, medium and high on a scale of 0 to 1 might be represented by 0.4, 0.6 and 0.8. In a catalytic reaction where Gli1 is a potent non-stoichiometric activator, a concentration of 0.001 might be "high" in that it causes the reaction to achieve its maximum rate. This information can be put into the model rules if available. If not, model simulations can still be run using whatever information is available.

### Common motifs

#### Positive and negative feedback

Feedback motifs are common in biological systems [39-43]. Negative feedback loops are essential for maintaining equilibrium and resisting change while positive feedback structures amplify signals to enable switch-like behaviour [39]. Gli1 is a transcription factor that promotes transcription of Ptc1 [28,30,33,44]. Figure 2 highlights the Gli1 feedback loop in the hedgehog pathway. NmycP and the transforming event required for tumor growth are constitutively "on" in figure 2 simulations in order to isolate the effects of Gli1 feedback on Ptc1 and tumor growth. When Shh signalling is active, Ptc1 is repressed, causing increased Gli1 expression. When the Gli1 feedback loop is active, as in figure 2.a, the system is self-regulating and tends toward an equilibrium level of Ptc1, Smo and Gli1, as increased Ptc1 expression tends to upregulate Gli1 even in the presence of Shh [33]. When the feedback loop is broken, as in figure 2.b, Shh signalling causes rapid suppression of Ptc1 to low levels and growth of Smo and Gli1 to high levels. Disconnecting the feedback loop is accomplished in the model by simply removing Gli1 as a reactant from the production reaction for Ptc1 (rule 3 in figure 1).

#### Feedforward motif

**Figure 3:**
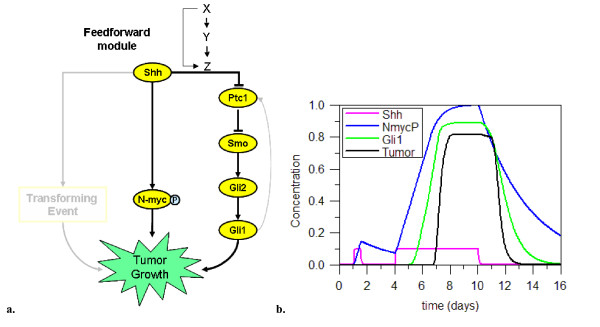
a. A feedforward motif in the hedgehog pathway is highlighted. b. To isolate the dynamics of the feedforward motif, the transforming event was made constitutively "on" and the Gli1 feedback loop to Ptc1 was deleted. A short, half-day upregulation of Shh was given at the start of day 1, then turned off. Though NmycP responded quickly to this transient signal, it was not sufficient to activate Gli1 transcription. Shh was upregulated for a longer period starting on day 4. After a one day delay, Gli1 transcription commences. Tumor growth begins when Gli1 reaches a moderately high level. When Shh transcription ends, NmycP levels begin to drop immediately, but Gli1 decline is delayed. Thus, a series of prolonged aberrant Shh activation events could sustain tumor growth.

Cells have a remarkable ability to orchestrate precise sequences of events using imprecise components. An important mechanism for accomplishing this feat is to filter out some signals and respond to others. A feedforward motif can filter the effects of transient signals while allowing sustained signals to activate downstream components [45]. Figure 3 isolates a feedforward motif in the hedgehog pathway. To isolate the dynamics of the feedforward motif, the transforming event was constitutively "on" and the Gli1 feedback loop to Ptc1 was deleted. Shh production was on for one-half day at the start of day 1, and then turned off. Though NmycP responded quickly to this transient signal, it was not sufficient to activate Gli1 transcription. On day 4 a longer Shh signal was turned on. Gli1 transcription began approximately one day after Gli1 production began. Tumor growth begins when Gli1 reaches a moderately high level. When Shh transcription ends, NmycP levels begin to drop immediately, but Gli1 decline is delayed. Thus, a series of prolonged aberrant Shh activation events could sustain tumor growth. Note that times and rates in this example were implemented to illustrate modeling concepts and, though physiologically reasonable, may not be accurate.

The feedforward system in figure 3 offers a potential explanation for the occurrence of medulloblastoma in haploinsufficient Ptc^+/- ^mice. Approximately 14–20% of heterozygous Ptc^+/- ^mice develop medulloblastoma tumors, whereas these tumors are rare in Ptc^+/+ ^mice [33,34]. Lower production of Ptc1 in heterozygous mice may make the probability that a perturbation to the hedgehog pathway will result in decreased Ptc1 expression for a long enough time to allow tumor growth to initiate. Transient Shh upregulation, as seen in the simulation, is not sufficient to allow sufficient Gli1 increase if Ptc1 production is high.

#### Single Input Motif (SIM)

**Figure 4:**
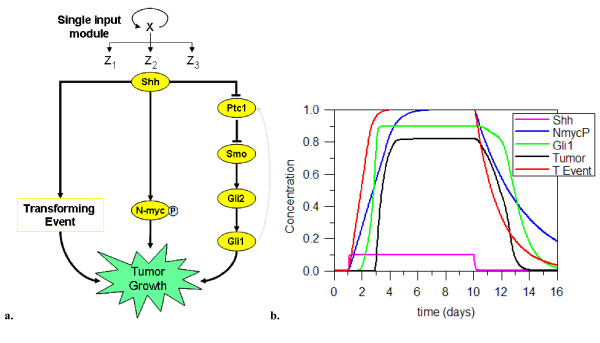
Shh activates three separate pathways in this model, each with a different activation and decay threshold and rate. a. A single input motif in the hedgehog pathway is highlighted. b. Simulation reveals that the unknown "transforming" event activates immediately when the Shh signal reaches a high level. NmycP production is also activated immediately by Shh, but it rises more slowly. Gli1 production is delayed by the longer signaling chain and threshold levels of Smo and Gli2 that are required for transcription. Tumor growth requires activation of all three pathways at a sufficient level. When the Shh signal is removed, rapid decay of the transforming event and NmycP cause tumor growth to quickly stop. Gli1 levels are maintained for a short delay before declining after the Shh signal is turned off.

In a related manner, temporal coordination of developmental processes can be achieved by differential response to a common signal [45,46]. Single input motifs involve activation of several parallel pathways by a single activator [45]. In figure 4, Shh activates three separate pathways. Each of these parallel pathways is required for tumor growth and each has a different activation threshold, decay threshold and rate. The rules for this simulation are as for the basic model in figure 1, with the Gli1 feedback loop deleted to illustrate this motif. The simulation curves have the same shape and characteristic response profile as the differential equation simulations in [45], demonstrating that intuitive rules define the correct dynamic behaviour.

#### Chemical reaction kinetics

Although analytical equations are known for basic reaction kinetics and approximations for enzyme-catalyzed reactions, it is desirable to use simple rules to describe such reactions when the goal is to integrate these with other processes for which analytical equations are not known. A number of software packages are available for modeling reaction dynamics with differential equations and can be found, for example, on the systems biology workbench site [47]. Generic numerical software that might also be suitable can be found in a number of places, including the netlib numerical software library [48]. Rule-based representations of chemical reactions are relatively simple to write and easily modified, which is useful when exploring the system effects of different possible reactions through simulation.

Because of the universal approximation properties of fuzzy logic models, they are able to represent any nonlinear process as accurately as desired, given sufficient data. It may be easier to make very precise chemical reaction models with differential equations, but robust system models that integrate many levels, from approximate cellular chemistry to clinical data, may be more useful for some medical research and more easily constructed with linguistic rules by biologists and clinicians without numerical modeling experience.

Rules to determine reaction rates from substrate concentrations can be determined in a variety of ways. The mathematical details for fuzzy logic rules most appropriate for modeling chemical kinetics remain to be derived. For modeling biological systems, simplicity with reasonable accuracy is a high priority. A second order reaction and an enzyme catalyzed reaction, both involving activation of Smo by Ptc1, are implemented with fuzzy rules and examined here.

An essential part of hedgehog pathway activation involves release of Ptc1 repression of Smo. Smo activates downstream transcription factors of the Gli family, which in turn upregulate cell cycle components [31,33,38,49]. One model suggests that Smo is in a balance between active and inactive states. Smo is activated when it combines stoichiometrically with a small molecule (sm) that changes its conformation. The small molecule must be actively transported across the cell membrane by Ptc1, which acts catalytically.

**Figure 5:**
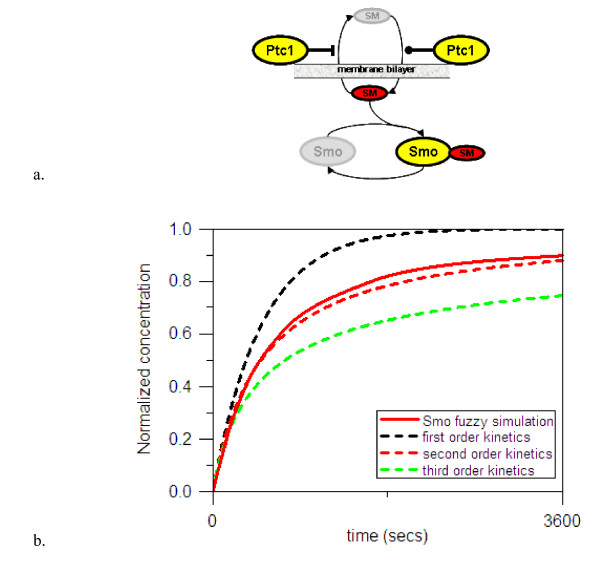
a. Smo is activated when it combines stoichiometrically with a small molecule (sm, red) that changes its conformation. The small molecule must be actively transported across the cell membrane by Ptc1, or is prevented from exiting the cell membrane by Ptc1, which acts catalytically. Active forms are in color, inactive in gray. b. A time series for activated Smo concentration is shown when inactive Smo combines stoichiometrically with a small molecule activator. This is a second order reaction. Analytic solutions for first, second and third order kinetics are also shown. Rules for the model is simulated are described in the text.

Figure 5 shows a model of Smo reaction with a small molecule agonist in a second order reaction after transport facilitated by Ptc1. Analytical solutions to first, second and third order kinetics are compared to simulations from a rule-based model for Smo activation by a small molecule. The simulated time series for Smo activation follows second order kinetics quite closely. Analytic solutions for first, second and third order kinetics are shown for comparison. This is intended to show that relatively simple rule-based models give reasonable biochemical dynamics. We note here that the default rules for first order reactions, where the rate is proportional to substrate concentration only, is mathematically equivalent to the usual differential equation for a first order reaction. Third order reactions are also well-approximated by simple reaction rules with three substrates.

The model in figure 5 is simulated with the following rules, with only three essential reactions. For each reaction, take the harmonic mean of substrate concentrations (zero/v. low/low/med/high/v. high); then reaction rate is (zero/v. low/low/med/high/v. high). The three reactions are: Reaction 1: Smo_inactive and sm are substrates and active Smo is the product. The rate coefficient is 1.0e-3 s^-1^. Reaction 2: the reverse reaction, has one substrate, activated Smo-sm complex, and two products, with the rate coefficient 1.0e-3 s^-1^. Reaction 3: The sm is transported across the membrane at a rate much faster than it is used by Smo and is continually supplied at a constant rate that is catalyzed by Ptc1.

**Figure 6:**
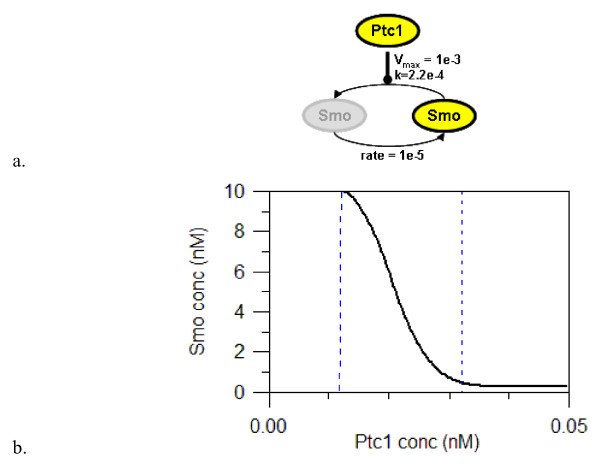
Smo inactivation catalyzed by Ptc1. In this simulation two reactions are used, one for activation from a precursor and one for deactivation of Smo. Activation occurs spontaneously at a constant rate. The inverse deactivation reaction is catalyzed by Ptc1 and is much faster when sufficient Ptc1 is present. Here Ptc1 is an essential catalyst: in its absence the deactivation reaction does not occur and Smo is continuously activated and maintained at a high level. Smo concentrations were set to be in the range of 0.0 to 10.0 nM. The concentration of Ptc1 is given as a fraction of Smo concentration. The vertical dashed lines indicate the concentration range over which the maximum inactivation rate is attained. The rules for this model include only two reactions. Reaction 1: Smo activation is proportional to the substrate concentration, Smo_inactive. The product is Smo and the rate constant is 1.0e-5. Reaction 2: Smo (active) is the substrate, Smo_inactive is the product, and Ptc1 is an activator. The rate is computed by harmonic averaging of the outcomes for the following rules: the rate is proportional to Smo concentration; the rate is zero when Ptc1 is zero or very low, and very high when Ptc1 is low or greater.

To analyze the system dynamics of the hedgehog pathway, it may be sufficient to model the catalytic nature of Ptc1 action heuristically using rules that capture the nonlinear effects of Ptc1 on Smo: if Ptc1 is zero, Smo activation is at its normal or highest level, V_max_. When a small amount of Ptc1 is present, Smo concentration is only 20% of its highest level (a linguistic value of "very low"). If Ptc1 is higher than that, the equilibrium concentration of active Smo goes to near zero. Normally, the linguistic terms zero, very low, low, medium, high and very high are assumed to mean fractions 0.0, 0.2, 0.4, 0.6, 0.8, 1.0, respectively, of the maximum possible value. These are used as defaults for all rules. However, these can be adjusted when more information is available. In figure 6, Ptc1 catalyzes Smo inactivation. It was reported that a concentration of Ptc1 of 1/45 the concentration of Smo reduces the level of active Smo by 80% [50]. The meaning of very low, low and medium we set at 0.012, 0.022 (1/45) and 0.032 for Ptc1 when it acts enzymatically to deactivate Smo. The rule for this reaction was: the inactivation rate is zero when Ptc1 is zero or very low and very high when Ptc1 is low or higher. The curve for equilibrium Smo versus Ptc1 concentration in figure 6.b shows the typical S-shape for an enzymatic reaction, with the greatest change in rate occurring for Ptc1 concentration of "low", the value at which the rate goes from zero to very high.

It is important to emphasize that the goal here is not just to show that fuzzy rules can be found to match differential equation models of chemical kinetics. Rather, a model based on intuitively reasonable rules that describe the reaction qualitatively produce reaction dynamics that are very close to dynamics derived from chemical kinetics using rigorous mathematical derivations.

### Oscillators, switches and discrete events in the cell cycle

Cell cycle machinery plays a central role in cell proliferation, apoptosis and cell fate determination. Failure to exit the cell cycle at the correct time may cause abnormal development and aberrant re-entry into the cell cycle by terminally differentiated cells may be a primary cause of many cancers. Understanding regulation of the cell cycle is therefore a central issue in the application of molecular biology to medicine. For that reason, a number of differential equation models of the cell cycle in yeast have been constructed for research purposes [51-54]. Regulation of the mammalian cell cycle is more complex than that of yeast, though many homologous genes and proteins and common network motifs are involved.

A model of the mammalian cell cycle was constructed with several important components. Chromosome separation and cell division were included as discrete events that occur when certain conditions are met. The cell cycle is an oscillatory system that involves not only continuous processes, but a series of discrete events [55]. Discrete events are an important mechanism in cells for controlling the precise timing of key events and insuring irreversibility with imprecise components [56,57]. Thus, even though many cell cycle parameters are poorly constrained, the overall behaviour of the cell cycle system is well characterized and heuristic modeling approaches are suitable for studying the control dynamics or testing possible ideas for new drug targets [58].

**Figure 7:**
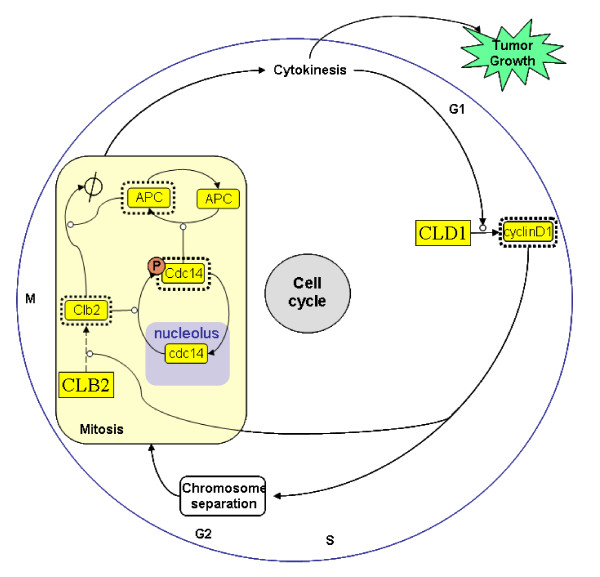
The cell cycle model shown integrates chemical kinetics, enzyme catalyzed reactions and discrete events, such as chromosome separation, cell division, and entry/exit to a combined G1SG2 phase. Tumor growth is a process that is linked explicitly to the cell cycle, thus providing a quantitative model that connects a molecular process model to a clinical manifestation. Each cell division increments tumor size, while a continuous apoptotic reaction causes slow decay of tumor size. Rules for this cell cycle model are given in the text. Simulation results are presented in the succeeding figures.

Figure 7 presents a cell cycle model that illustrates how chemical kinetics, enzyme catalyzed reactions and discrete events, such as chromosome separation, cell division, and entry/exit to a combined G1SG2 phase can be integrated. The primary molecular components of this model are cyclinD1, cyclinB2, APC, and Cdc14. Cell cycle models with these or similar components have been constructed using differential equations [54,59].

Tumor growth is linked to this cell cycle via cell division. Tumor size in this case is a measure of the number of cell divisions that have occurred. If the cell cycle arrests, tumor size shrinks slowly through a continuous apoptotic reaction. Rules for this cell cycle model are as follows:

1. cyclinD1 production is continuous

2. cyclinD1 decay is proportional to cyclinD1 and APC levels and is rapid

3. cyclinB production is proportional to cyclinD1 level

4. cyclinB decay is zero when APC is less than high, is low when APC is high, and is very high when APC is very high.

5. Cdc14 production is proportional to cyclinB levels

6. Cdc14 decay is continuous and proportional to Cdc14 concentration

7. APC production is proportional to Cdc14 level, but is zero when Cdc14 is very low and low.

8. APC decay is directly proportional to cyclinB, cell mass and entry into G1SG2 and determined by the harmonic average of these.

9. Chromosome separation event occurs when cyclinD1 is medium or higher and G1SG2 is in the on state (G1SG2 is a discrete event, either on or off). G1SG2 turns off when chromosome separation occurs.

10. Cell division occurs when cyclinB is low or less and decreasing at any rate; chromosome separation event must also have occurred. G1SG2 turns on when cell division occurs and chromosome separation turns off.

11. Cell division turns off when cell mass is very low or less and cell division is currently on.

12. Cell mass growth is constant. When cell division occurs (a single event), cell mass is halved. Cell mass has a range of values between one and two.

**Figure 8:**
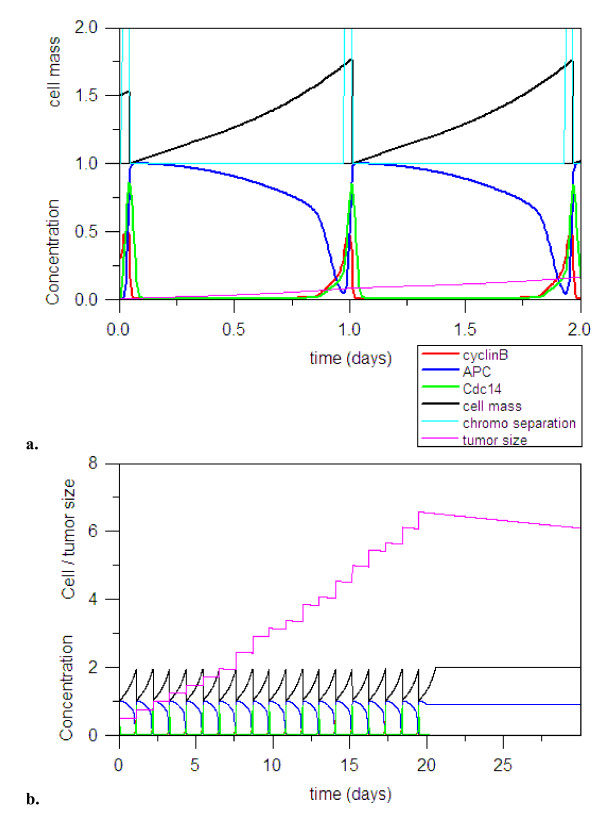
Simulations of the cell cycle from figure 7 using a rule-based model. a. The growth rate of cell mass has a controlling effect on the rest of the cycle. It was set to make the cycle length approximately 1 day, a typical value for human cells undergoing mitosis [60]. The shape and ordering of curves for cyclin B, Cdc14 and APC are very similar here to those shown in Tyson's cell cycle model [59]. The cell mass ranges between 1.0 and 2.0. Chromosome separation is a discrete event that is either on or off. For clarity, the curve was shifted up by one unit in the graph. Another discrete event, cell division, is not shown here. b. Tumor growth in this model is controlled directly by the cell cycle. The rule is: tumor growth is incremented whenever cell division occurs. Thus, if the cell cycle stops, tumor growth stops. Tumor growth is also faster when the cell cycle period is shorter. Tumor cell apoptosis occurs at a constant rate. Tumor size has a large range of values, 0 to 100 in arbitrary units, to allow virtually unlimited tumor growth in model runs.

The growth rate of cell mass has a controlling effect on the rest of the cycle. It was set to make the cycle length approximately 1 day, a typical value for human cells undergoing mitosis [60]. Figure 8 shows time courses for molecular components and events in the cell cycle model, as well as tumor growth. The shape and ordering of curves for cyclinB, Cdc14 and APC are very similar here to those shown in Tyson's cell cycle model [59]. Chromosome separation is a discrete event that is on for a brief time at the beginning of mitosis and then turns off when cell division occurs. Another discrete event, cell division, is not shown here. If either of these events does not occur (turn on), the cell cycle halts. Similarly, knocking out any of the critical components of this pathway, cyclinD1, cyclinB, Cdc14 or APC, for example by deleting their production reaction, will also cause the cycle to halt. In figure 8.b the cell cycle was set to stop suddenly on day 20. When this happens, tumor growth, a measure of the number of cell divisions that have occurred, stops and the tumor slowly decays through the apoptotic reaction. The latter is a simple reaction with tumor size as the sole participant (as a substrate that is used up).

### Modeling and drug target discovery

Computational models are potentially very useful for exploring the effects of manipulating complex pathways through drugs or other means. Rule based models of two features that make them promising tools for drug target discovery. First, it is easy for experts to use their domain knowledge to build models. Rules can be changed easily without mathematical manipulations to carry out thought experiments with many hundreds or thousands of components. Secondly, algorithms for automatically manipulating fuzzy rules have been developed for a variety of engineering applications and include genetic algorithms and neural nets. It may be possible for experts to build first-stage models of pathways and then apply computational methods to optimize the models using other data sources, or to search for optimal drug targets.

To illustrate the use of the model for carrying out computational experiments a rule-based model was constructed using elements of the previously presented pathways. Our goal in this example is to explore the effects of a drug, HhAntag, that reportedly interferes with Gli1 activation of cyclinD1, thereby stopping progression of the cell cycle [34]. HhAntag was introduced as a new component to the model in figure 1. HhAntag inhibits both Gli1 and, to a lesser extent, Gli2 expression downstream of Smo.

**Figure 9:**
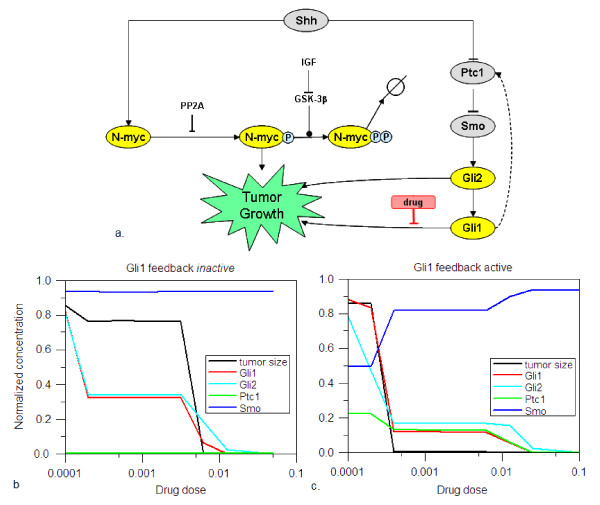
a. This model is similar to the one in figure 1, but a few more details concerning N-myc activation are added. The transforming event of figure 1 is constitutively on when Shh is high, so it is not shown here. Tumor growth requires Nmyc-P and either Gli1 or Gli2 to be active. b. the feedback loop shown as a dotted line from Gli1 to Ptc1 is inactive. The drug suppresses Gli1 and Gli2 activity downstream of Smo, inhibiting tumor growth, but high Smo levels allow continued expression of Gli2, which is able to activate the cell cycle. Only when high drug doses are present are Gli1 and Gli2 sufficiently suppressed to stop tumor growth. c. The feedback loop is now active; Gli1 and Gli2 are suppressed by the drug, but small levels of Gli1 are sufficient to activate Ptc1 production, supressing Smo expression, hence Gli2 is lower. As drug dose increases, Gli1 is suppressed and eventually Ptc1 production stops, allowing Smo to be expressed.

An interesting reported effect of HhAntag on cultured neural tissues is that a relatively low dose suppresses Gli1 levels, but a thousand fold increase is needed to halt tumor growth [34]. This effect is not observed *in vivo*. An important key to this difference is the suggestion that the feedback loop from Gli1 to Ptc1 is operates in the cultured system but not *in vivo*. As discussed previously, Ptc1 acts catalytically on Smo expression: very low levels of Ptc1 are sufficient to significantly reduce Smo activity [29]. As shown in figure 9, when the Gli1 feedback loop to Ptc1 is active small doses of drug are sufficient to suppress tumor growth (figure 9.b). Without this feedback, the drug is not as effective in low doses. Only at much higher doses can the drug suppress both Gli proteins sufficiently to stop tumor growth (figure 9.c).

It should be emphasized here that the hedgehog pathway is not well understood and the model presented here is in preliminary stages. Nevertheless, it illustrates important modeling ideas. The next step is to replace the tumor growth module with a cell cycle model. First, we develop and test a preliminary cell cycle model using rules. This will then be connected to the hedgehog pathway.

**Figure 10:**
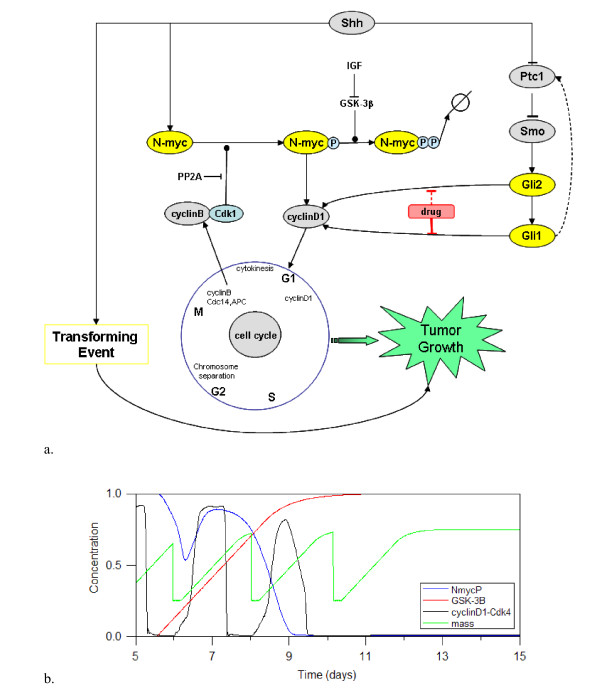
In this model, the tumor growth process is replaced with the cell cycle model from figure 7. Components of the hedgehog pathway now interact directly with cyclin D1; mitotic cyclin (cyclin B) is required for Nmyc phosphorylation to the active Nmyc-P state. b. If the cell cycle is activated by injecting Nmyc-P initially, the effect of other components on the cycle can be studied. In this figure, IGF is decreased beginning on day 6, allowing GSK-3β to increase. Nmyc-P is then phosphorylated and degraded. The effects of drug dose will be the same for this model as in figure 9, as the primary action of the drug on Gli2 has not been changed. However, adding details to the cell cycle now allows more detailed investigation of the interaction between the drug, its targets, and cell cycle components.

A more complete model for hedgehog regulation of the cell cycle is shown in figure 10. The cell cycle model discussed previously has been combined with the components in figure 9. Integration of these rule-based models was simple and required little more than pasting the rule files for each into one file and modifying the reactions for cyclinD1 so that Gli1 and Gli2 regulated its production. In this model, Shh directly induces Nmyc expression and indirectly affects Nmyc posttranslational modification, mediated by its indirect targets, cyclinB and possibly other cyclins [30,44,61]. Shh also binds to Ptc1, as described previously, activating Gli expression. The tumor growth process from figure 8 is now replaced by the cell cycle model from of figure 9. Note that a circular pathway exists among activated cyclinB, Nmyc and its phosphorylated form NmycP, and cyclinD1. An additional component or transforming event, mentioned in figure 1, is needed to start this cycle, which reinforces the suggestion that another yet unknown target may also be required for cell transformation [61].

Once started, the cycle will continue as long as the Shh pathway remains active. Mitotic degradation of Nmyc permits neuronal precursor cell cycle exit in the absence of Shh signalling or in the case of an intrinsic program-directed shift toward differentiation. Thus neural fate specification and cancer activation are regulated by the same machinery.

In figure 10.b simulation time courses show the effect of decreasing insulin growth factor (IGF) on the cell cycle. In this figure, IGF is decreased beginning on day 6, allowing GSK-3β to increase. Nmyc-P is then phosphorylated and degraded. Note that Nmyc-P initially decreases, and then recovers for one cell cycle through complicated feedbacks. Incorporating as many of these complicated interactions into a model is essential for identifying potential drug targets, as cells are remarkably robust and have built-in fault tolerance systems that may not be evident from examination of static network or interaction diagrams [56,62,63]. The effects of drug dose will be the same for this model as in figure 9, as the primary action of the drug on Gli2 has not been changed. However, adding details to the cell cycle now allows more detailed investigation of the interaction between the drug, its targets, and cell cycle components.

## Conclusion

An important goal of computational modeling of biochemical networks is to identify potential drug targets *in silico*. Computational tools that can enhance the ability of researchers to reason through the complex dynamics of pathways of interest may be useful for researchers trying to keep track of dozens or hundreds of interacting network components, while automatic methods for manipulating pathway models to search for drug targets have the potential to revolutionize the drug development process [64].

Integrating the rapidly growing quantities of high throughput data into meaningful pathway information is a bottleneck to computational drug screening and discovery [65]. Although informatics tools are currently being used to identify potential pathway interactions and putative targets, target identification necessarily requires detailed understanding of the structure of the pathway and the role of specific genes and proteins. This information is generally available in journal articles as linguistic pathway descriptions or interactions derived from hypothesis-driven experiments [66].

A major advantage of the modeling approach presented here is that it enables the incorporation of biological expertise into the modeling process. At the same time, it does not prevent integration of multiple high throughput data sources into the model. New evolutionary algorithms based on statistical estimation, called estimation of distribution algorithms [15,67], enable Bayesian probability distributions to be used as input data to optimize system parameters. Such statistical data is commonly available from bioinformatics analyses of microarray expression data ([68-70]) or proteomics data [71,72]. The framework presented by the fuzzy logic model presented here thus allows representation of expert knowledge about a pathway, and integration of other sources of numerical data with that data. Further research into efficient algorithms for integration of data from many sources is an important area for further research.

Drug discovery is a difficult challenge for computational and systems biology. Finding a "clever way to throw away the details may be the most important part of model building" [1]. Intelligent hybrid systems are an important method for modeling dynamic networks and complex biological processes with linguistic or qualitative-logical data, while allowing for more accurate models as more data becomes available.

The initial motivation of this modeling approach was to shift the burden of modeling to biological description and biological data. We deliberately sought to develop a modeling paradigm that imitates the logical reasoning that biologists use when analyzing a complex pathway diagram to understand its function and develop new hypotheses to explain observations.

Another motivation for this work, which will be developed and explicated in future publications, was to find a quantitative representation of biological systems that was simple enough to be manipulated by genetic algorithms as a possible approach to computational drug target discovery and for integration of multiple data sources. Such a program will likely be similar to current engineering approaches to fuzzy system discovery [3,4,9,10] and will be a subject for future research.

## Methods

Fuzzy logic (FL) provides a simple way to arrive at a definite conclusion based upon vague, ambiguous, imprecise, noisy, or missing input information. FL's approach to control problems mimics human reasoning, only much faster. A FL biochemical model requires some numerical parameters in order to operate, such as initial values and rate coefficients, but exact values of these numbers are usually not critical. Since dynamic rules are defined in terms of fuzzy quantities, the logic of the dynamics can be prescribed without precise numerical values.

### Bionet implementation of fuzzy network models

A potential drawback of fuzzy logic models is the rapid growth of the rule table when many inputs are involved. Biological networks typically involve hundreds or thousands of interacting variables, so this is a serious concern. If the number of fuzzy sets used for each variable is F, then the number of rules required to define rules for all possible inputs is F^N^, where N is the number of variables. At least four fuzzy sets are needed; even a few hundred variables causes the number of rules needed to specify a dynamic model to become huge.

Fortunately, a several simplifications are possible when modeling biological networks that greatly reduce the size of the rule base, even when hundreds or thousands of variables are involved. Most importantly, the number of variables involved in any reaction or process is relatively small. Typically only a few species are involved in a reaction; the vast majority of proteins or genes do not interact appreciably. Thus, a biological system can be modelled as many networked reactions or processes.

**Figure 11:**
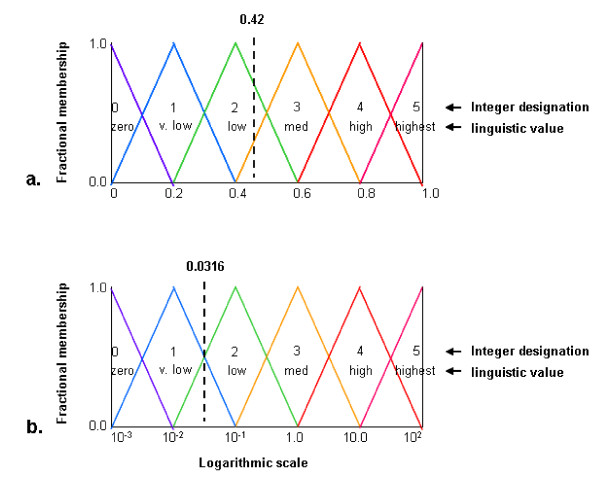
a.Triangular membership functions on the interval 0 to 1. Linguistic values and equivalent integer designations are shown with each membership function. Centroids for the triangular functions evenly divide the interval into five segments. All examples in this paper used these membership functions, except that the length of the interval was varied in some cases, or the centroids were adjusted. An example of fuzzification of the real value 0.42 is: 0.42 = 0.9 (0.4) + 0.1 (0.6) = 0.9 low + 0.1 medium. B. triangular membership functions are linear on the log of the values. For example, if the range is [10^-3^, 10^2^], then 0.0316 = sqrt(0.001) is fuzzified by linearly combining logarithms of the fuzzy set values. Log(0.0316) = -1.5 = 0.5 log(10^-2^) + 0.5 log(10^1^) = 0.5 very low + 0.5 low.

Figure 11 illustrates a network of interacting fuzzy logic models. For simplicity these models that consist of networked fuzzy biological processes will be called of Bionet models. The structure is similar to a functional hybrid Petri net [73], with processes replaced by fuzzy inference models. The method here is distinguished from functional Petri nets by the rule based process model. The focus here is on biochemical networks, so variables are generally protein concentrations or gene expression levels. However, Bionet variables are completely general and may represent any quantity. This is quite useful when building models in a hierarchical fashion. A variable might represent an entire complex pathway or tissue. Rules then define how the "blackbox" pathway or tissue interacts with other variables without reference to internal complexities. Later, mechanistic details can be added as more data becomes available.

In general, the number of rules needed for each reaction is equal to the product of the number of fuzzy sets for each component involved in the reaction. If each reactant has six fuzzy sets or states and there are four reactants in a process, then there are 6^4 ^= 1296 rules that must be specified. Computations are simplified further by noting that any real number belongs to at most two fuzzy sets. Thus the number of rules that must be evaluated at each time step is 2^4 ^= 16 when there are four reactants. Some heuristics can be used to automatically compute the rules. In general, reactions are limited by the minimum rate for any one of the reactants. If a substrate for a reaction has concentration zero, for example, that will limit the rate of reaction regardless of how much of any of the other reactants is present. Similarly, if a strong inhibitor is present at its highest concentration and it shuts down the reaction completely, then it does not matter what the other concentrations are. With similar heuristics, it is sufficient to specify the effect of each component individually on the reaction. Rules for combining reactants are then determined automatically by applying appropriate averaging heuristics. Example simulations below illustrate the input information required.

### Membership functions

All simulations in this paper used six fuzzy sets, corresponding to the linguistic variables zero, very low, low, medium, high and highest. The centroid locations for each of these sets, a scale of 0.0 to 1.0, are 0.0, 0.2, 0.4, 0.6, 0.8, and 1.0. When the universe of discourse is other than 0 to 1, centroids are the endpoints and fractional increments of 0.2 define the other centroids. We found that six fuzzy sets were adequate for all simulation requirements presented here. The discretization error in numerical differential equations can be reduced by using a finer grid or by using a variable grid that puts finer grid points where greater accuracy is needed. The standard additive model can achieve greater flexibility for approximating functions in a similar manner, either by increasing the number of fuzzy sets or by moving the centroid locations. The latter was illustrated earlier with the example of catalytic activation of Smo by Ptc1. The centroids for very low, low and medium were defined by 0.0012, 0.0022 and 0.0032, respectively, while leaving the other centroids unchanged. In Bionet, default centroids can be redefined by simply listing the centroid values after the rules for a variable in a reaction. Note that the meaning of a linguistic variable such as "very low" is reaction dependent. When a protein is a stoichiometric substrate in a reaction, the default centroids might be appropriate; while for the same variable acting as a catalyst the meaning of very low might be quite different.

### Model specification

Input information for a Bionet model consists of a list of variables, also called nodes, along with initial values. Each reaction between variables is a fuzzy rule-based inference. A reaction consists of one or more variables and a reaction rate constant. The rate constant scales the reaction rate that is determined as an output from the fuzzy inference and is 1.0 by default. Logical pathway models can be built to test the logic of interactions even if little is known about rate constants. If all reactions are defined to have only two output states, highest and zero, or on and off, the entire network is equivalent to a dynamic Boolean network [74,75].

Variables play different roles in different reactions. A variable may be a product in a synthesis reaction. In other reactions it may be an inhibitor or activator – or even both depending on its concentration. For simplicity, default rules are defined for four basic roles: substrate, product, activator and inhibitor. Stoichiometry determines how quickly the reactant is produced or used up; activators and inhibitors have stoichiometry of zero in a reaction.

The essence of model dynamics is the fuzzy inference that maps fuzzy input variables to fuzzy output variables. Conversion of precise real values to fuzzy or linguistic values is accomplished by fuzzification. This is illustrated in figure 11. The domain or universe of discourse for a variable can be defined by any real numbers. The minimum and maximum values are constants for the whole simulation. The default range is 0 to 1, since in many cases we are interested in network dynamics with normalized values. However, specifying a different range is trivial, requiring only that the range be specified when the node and its initial value are declared. Most importantly, reaction dynamics are defined in terms of fuzzy sets, not the real values that are attached to those sets by the centroid values. In figure 11.a the domain is divided into six fuzzy sets of equal size. This is the default, though it can also be changed by listing the centroid values with the node declaration. Although the endpoints are fixed, the placement of intermediate centroids may be context dependent and differ with each reaction. This reflects the fact that the meaning of linguistic terms such as "low" may depend on whether a particular molecule is a product or activating enzyme, for example. An example of fuzzification of the real value 0.42 is: 0.42 = 0.9 (0.4) + 0.1 (0.6) = 0.9 low + 0.1 medium.

For wider dynamic range, a logarithmic scale has also been implemented. As illustrated in figure 11.b, triangular membership functions are linear on the log of the values. For example, if the range is [10^-3^, 10^2^], then 0.0316 = sqrt(0.001) is fuzzified by linearly combining logarithms of the fuzzy set values. Log(0.0316) = -1.5 = 0.5 log(10^-2^) + 0.5 log(10^1^) = 0.5 very low + 0.5 low. Logarithmic scales are useful when concentrations vary over a large range and changes in biochemical activity require concentration changes over many orders of magnitude. The rate at which the actual (real-valued) concentration changes in each reaction is still determined by the reaction rate. We emphasize again that the reaction rules are still defined in terms of the effect of fuzzy values (low, medium, and so on) on the reaction rate. The fuzzy set definitions, whether linear or logarithmic, provide meaning to the linguistic fuzzy sets in the context of particular reactions.

### An example

**Figure 12:**
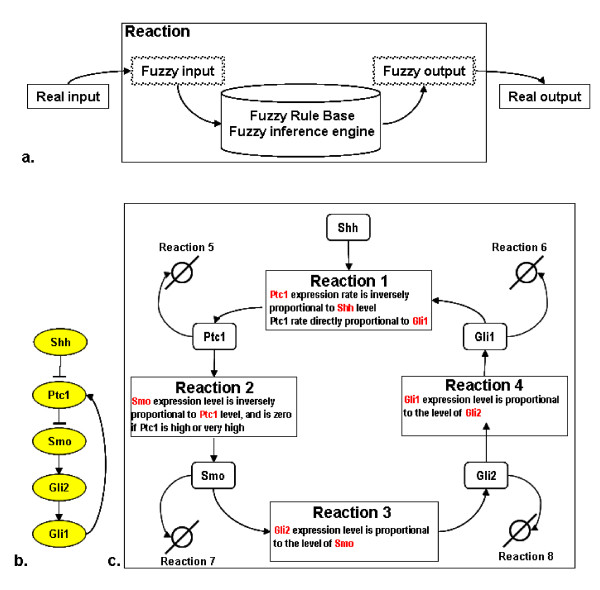
a. Each reaction or process is a fuzzy inference system, with real inputs and real outputs. b. A subset of the hedgehog pathway is represented here. c. The pathway in b. is now represented as a network of fuzzy inference systems. Reactions are linked together by variables that may participate in more than one reaction. At every time step, all reactions are executed and variables are updated.

The reaction network in figure 12 consists of 5 variables and 8 reactions. Four reactions are not shown – each variable except Shh decays at a constant rate. Shh is set to an initial value that doesn't change. Simulation results for this example were shown in figure 2. The universe of discourse or range for each variable and an initial value are set for each variable. The input file used is:

Node Shh 0.1 log 0.001 0.1

Node NmycP

Node Ptc1 0.5

Node Smo

Node Gli1

The keyword 'Node' identifies this as a node definition. Initial values 0.0 by default if not given explicitly. Similarly, the domain or universe of discourse is [0,1] if not given. In the above, Shh has an initial value of 0.1 and a domain of [0.001, 0.1] on a logarithmic scale. This was done primarily for illustration, since in this simulation Shh was set to a constant value and maintained. The initial value for Ptc1 is 0.5; all others have initial value 0.0. The meaning of "low" or "high" for each variable is context dependent and is defined for each reaction that a variable participates in.

The model has 8 reactions, which are essentially fuzzy inference engines that map the fuzzy input variables to a fuzzy output rate. The network of reactions is illustrated in figure 12. Reactions rates are computed and fire on each time step. The rate is scaled by the rate constant to give a reaction rate at this time step. Each reaction consists of a reaction name, a rate coefficient, and a list of participating reactants. The reactants can be used up (substrates, stoichiometry < 0), produced (products, stoichiometry > 0) or participate without changing concentration (activator or inhibitor, stoichiometry = 0). The reactions for Smo production and decay, Reactions 2 and 7, are shown here:

Reaction Smo_production 0.002

   pro Smo

   inh Ptc1 5 2 1 1 0 0

Reaction Smo_decay 0.002

   sub Smo

The keyword 'Reaction' defines a reaction definition. The reaction name follows the keyword. The number after the reaction name is the reaction rate constant. If not given the default value is 1.0. The second reaction shown here, Smo_decay, has a single reactant, Smo, which is a substrate. The 'sub' keyword implies the following default values:

Reaction Smo_decay 0.002

   sub Smo 0 1 2 3 4 5 stoi -1 centroids 0.0 0.2 0.4 0.6 0.8 1.0

The first six numbers that follow 'sub Smo' are the rules that define how the reactant Smo contributes to the rate of this reaction. The six integers are the output rate for each fuzzy level of Smo. These are a shorthand for the linguistic rules: "when Smo is zero, the rate is 0; when Smo is very low, the rate is 1 or very low; when Smo is low, the rate is 2 or low; when Smo is medium the rate is 3 or medium; when Smo is high, the rate is 4 or high; when Smo is highest, the rate is 5 or highest. If more information is available to give more accurate rules or centroids, they can be written here. More likely, these values will be set by automatic optimization methods when high throughput data is used to refine models that are initially built manually by experts. We have implemented a simple genetic algorithm that manipulates the rules and centroids. Incorporation of procedures for specific data sources remain for future research, but the model is designed for these enhancements.

The Smo_production reaction fires on every time step. The default rules for a product, Smo in this case, are 5 5 5 5 5 0. This means that the output rate is 5 or "highest" when the Smo concentration is zero through high (0 to 4). If Smo reaches the highest concentration, 5, it cannot go higher because that would exceed its defined limit. The rate goes to zero at that point. The inhibitor, Ptc1, has rules 5 2 1 1 0 0, which means that when Ptc1 concentration is high or highest, the rate is zero. Similarly, when Ptc1 is zero, the rate is 5 (unaffected), when Ptc1 is very low, the rate tends to low (2), when Ptc1 is low or medium, the rate is very low (1).

The actual reaction rate is computed as follows: the real concentration of each participating reactant is fuzzified. The rate is a multi-dimensional function of the number of reactants. Following the usual Standard Additive Model, the rate is found by adding fractional membership of each variable to find the total contribution:

∑s=05∑p=05f(s)f(p)Rule(Smo=s,Ptc1=p)=∑s=05∑p=05f(s)f(p)HarmonicAverage(Rule(Smo=s),Rule(Ptc1=p))     (1)
 MathType@MTEF@5@5@+=feaafiart1ev1aaatCvAUfKttLearuWrP9MDH5MBPbIqV92AaeXatLxBI9gBaebbnrfifHhDYfgasaacH8akY=wiFfYdH8Gipec8Eeeu0xXdbba9frFj0=OqFfea0dXdd9vqai=hGuQ8kuc9pgc9s8qqaq=dirpe0xb9q8qiLsFr0=vr0=vr0dc8meaabaqaciaacaGaaeqabaqabeGadaaakqaabeqaamaaqahabaWaaabCaeaacqWGMbGzcqGGOaakcqWGZbWCcqGGPaqkcqWGMbGzcqGGOaakcqWGWbaCcqGGPaqkcqWGsbGucqWG1bqDcqWGSbaBcqWGLbqzcqGGOaakcqWGtbWucqWGTbqBcqWGVbWBcqGH9aqpcqWGZbWCcqGGSaalcqWGqbaucqWG0baDcqWGJbWycqaIXaqmcqGH9aqpcqWGWbaCcqGGPaqkaSqaaiabdchaWjabg2da9iabicdaWaqaaiabiwda1aqdcqGHris5aaWcbaGaem4CamNaeyypa0JaeGimaadabaGaeGynaudaniabggHiLdaakeaacqGH9aqpdaaeWbqaamaaqahabaGaemOzayMaeiikaGIaem4CamNaeiykaKIaemOzayMaeiikaGIaemiCaaNaeiykaKIaemisaGKaemyyaeMaemOCaiNaemyBa0Maem4Ba8MaemOBa4MaemyAaKMaem4yamMaemyqaeKaemODayNaemyzauMaemOCaiNaemyyaeMaem4zaCMaemyzauMaeiikaGIaemOuaiLaemyDauNaemiBaWMaemyzauMaeiikaGIaem4uamLaemyBa0Maem4Ba8Maeyypa0Jaem4CamNaeiykaKIaeiilaWIaemOuaiLaemyDauNaemiBaWMaemyzauMaeiikaGIaemiuaaLaemiDaqNaem4yamMaeGymaeJaeyypa0JaemiCaaNaeiykaKIaeiykaKcaleaacqWGWbaCcqGH9aqpcqaIWaamaeaacqaI1aqna0GaeyyeIuoaaSqaaiabdohaZjabg2da9iabicdaWaqaaiabiwda1aqdcqGHris5aOGaaCzcaiaaxMaadaqadaqaaiabigdaXaGaayjkaiaawMcaaaaaaa@A58D@

If on a particular time step Smo is 0.55 = 0.25 low and 0.75 medium, while Ptc1 is 0.4 high and 0.6 highest, then equation (1) becomes

0.25 * 0.4 * *HA*(5,1) + 0.75 * 0.4 * *HA*(5,0) + 0.25 * 0.6 * *HA*(0,1) + 0.75 * 0.6 * *HA*(0,0)

= 0.1 * 1.7 + 0.3 * 0 + 0.15 * 0 + 0.45 * 0

= 0.17

All rates are computed on a 0 to 1 scale, and then scaled by the rate coefficient. The final rate for this reaction step is thus 3.4e-4. The value of Smo on the next time step will be 0.55034.

The list of variables and the definition of all reactions is the primary input to define a Bionet model. Start and end times for the simulation and the number of time steps are also needed as input. At each time step, all reactions take place and variables are updated. Bionet simulations are similar to explicit time-stepping schemes in numerical approximations to differential equations. Bionet implements the Standard Additive Model or SAM for each fuzzy reaction [4]. This implementation guarantees the universal approximator property of the model.

Time limits and time step size must be specified as for any dynamic model. This method uses explicit time stepping, so the step size is controlled by the fastest reaction. The condition rate*Δt < Δx_max _must hold for the fastest reaction, where xmax is either the difference between the highest and next-to-highest centroid for any variable that is a product substrate in that reaction, or x_max _is the difference between the next-to-lowest and lowest centroid for any substrate in that reaction. For all simulations in this paper, the time step was 1 minute. Most reaction rates were on the order of 10^-3 ^minutes, but the cyclin decay rates were faster (1.0 min^-1 ^for cyclin D).

Reaction order is asynchronous to prevent any bias in the simulation. The order of reactions is randomly chosen at every time step. Furthermore, if desired, a probability of firing may be assigned to a reaction. In this case, reactions will occur with some probability on each of the time steps. Models that include stochastic reactions can represent stochastic processes in cells, such as stochastic gene expression [76] or a probability of tumor formation. Whether or not this is an appropriate or useful model for stochastic biological processes remains to be determined and likely will depend on the particular phenomenon of interest. The flexibility of this approach allows for a variety of modifications to be tried in future research.

### Software availability and requirements

Simulation software, source code and input files containing rules for all examples discussed in this paper are available for download on the Bionet web site.

Project name: Bionet

Project home page: 

Operating systems: Platform independent; GUI version for MS-Windows only.

Programming language: java (Java 2 runtime environment)

License: Use of this software is governed by the Gnu General Public License [77].
